# A double-blind, placebo-controlled study of the short term effects of a spring water supplemented with magnesium bicarbonate on acid/base balance, bone metabolism and cardiovascular risk factors in postmenopausal women

**DOI:** 10.1186/1756-0500-3-180

**Published:** 2010-06-28

**Authors:** Richard O Day, Winston Liauw, Lynette MR Tozer, Patrick McElduff, Russell J Beckett, Kenneth M Williams

**Affiliations:** 1 St Vincent's Hospital, Clinical Trials Centre, Department of Clinical Pharmacology and Toxicology and University of NSW, Sydney NSW 2010, Australia; 2 Cancer Care Centre, St George Hospital, Gray St, Kogarah NSW 2217, Australia; 3 Datapharm Australia Pty Ltd, Drummoyne NSW 2047, Australia; 4 Unique Global Possibilities Medical Pty Ltd, Sydney NSW 2000, Australia

## Abstract

**Background:**

A number of health benefits including improvements in acid/base balance, bone metabolism, and cardiovascular risk factors have been attributed to the intake of magnesium rich alkaline mineral water. This study was designed to investigate the effects of the regular consumption of magnesium bicarbonate supplemented spring water on pH, biochemical parameters of bone metabolism, lipid profile and blood pressure in postmenopausal women.

**Findings:**

In this double-blind, placebo-controlled, parallel-group, study, 67 postmenopausal women were randomised to receive between 1500 mL and 1800 mL daily of magnesium bicarbonate supplemented spring water (650 mg/L bicarbonate, 120 mg/L magnesium, pH 8.3-8.5) (supplemented water group) or spring water without supplements (control water group) over 84 days. Over this period biomarkers of bone turnover (serum parathyroid hormone (PTH), 1,25-dihydroxyvitamin D, osteocalcin, urinary telopeptides and hydroxyproline), serum lipids (total cholesterol, HDL-cholesterol, LDL-cholesterol and triglycerides), venous and urinary pH were measured together with measurements of standard biochemistry, haematology and urine examinations.

Serum magnesium concentrations and urinary pH in subjects consuming the magnesium bicarbonate supplemented water increased significantly at Day 84 compared to subjects consuming the spring water control (magnesium - p = 0.03; pH - p = 0.018). The consumption of spring water led to a trend for an increase in parathyroid hormone (PTH) concentrations while the PTH concentrations remained stable with the intake of the supplemented spring water. However there were no significant effects of magnesium bicarbonate supplementation in changes to biomarkers of bone mineral metabolism (n-telopeptides, hydroxyproline, osteocalcin and 1,25-dihydroxyvitamin D) or serum lipids or blood pressure in postmenopausal women from Day 0 to Day 84.

**Conclusions:**

Short term regular ingestion of magnesium bicarbonate supplemented water provides a source of orally available magnesium. Long term clinical studies are required to investigate any health benefits.

**Trial registration:**

ACTRN12609000863235

## Background

Epidemiological observational studies have found an inverse association between the incidence of, and mortality from, cardiovascular disease and increased drinking of water containing mineral salts of calcium and magnesium [1-3]; particularly in women [4]. In case control studies, drinking water with greater than 8 mg/L of magnesium was associated with lower risk of death from myocardial infarction [4-6]. A recent meta-analysis of such epidemiology studies further supported the inverse association between magnesium levels in drinking water and cardiovascular mortality [7]. A lower risk of myocardial infarction among men drinking water with magnesium and bicarbonate with concentrations of greater than 110 mg/L compared to those who consumed magnesium in water with minimal bicarbonate was imputed from epidemiological studies in Sweden [6,8]. It is possible, therefore, that any beneficial effects from the magnesium content of waters may be enhanced by bicarbonate.

Clinical studies have reported that bicarbonate from mineral waters lowers bone resorption [reviewed in [9]] and may reduce cardiovascular risk as evidenced by associated decreases in early markers of atherosclerosis in healthy postmenopausal women [10]. Western diets generally have a high dietary acid load and are associated with in a low intake of minerals such as magnesium, calcium and potassium. Such diets can induce a chronic, low-level, metabolic acidosis [11-13]. The degree of acidosis also increases with age and, therefore, it has been postulated that acidosis may be associated with some of the diseases of aging [12]. Drinking water containing bicarbonate may represent a means of regulating acid base balance.

Magnesium is a cofactor for approximately 300 enzymes. About 50% of magnesium in the body resides in bone where it is directly involved in calcium and bone homeostasis. Magnesium affects the function of the parathyroid glands [14] where it acts as an agonist at the calcium-sensing receptors and, like calcium, can decrease parathyroid hormone (PTH) secretion [15]. Low magnesium status has been associated with cardiovascular disease, hypertension, diabetes [16] and osteoporosis [17]. Magnesium supplementation has been linked with suppression of bone turnover [18] and improvements in lipid profile and blood pressure [19,20].

This randomised double-blind, placebo-controlled study was conducted in postmenopausal women to investigate whether the short term regular ingestion of spring water supplemented with magnesium bicarbonate could contribute to the intake of magnesium and influence acid base balance, metabolic parameters of bone turnover and cardiovascular risk factors (blood pressure and serum lipids).

## Methods

A randomised, double-blind, placebo-controlled, parallel-arm study comparing the daily intake of 1500 mL - 1800 mL of magnesium bicarbonate supplemented spring water with non supplemented spring water (placebo) was undertaken in postmenopausal women, while on their usual diet, over an 84 day period. The study was conducted from 31 August 2005 to 3 November 2006 and approved by the St Vincent's Hospital Human Research Ethics Committee.

### Subjects

Ninety-one subjects attended the Clinical Trials Centre for screening for eligibility. All screened subjects provided written informed consent to participate in the study prior to commencement of screening procedures.

Only postmenopausal subjects aged 50 -70 years and with a body mass index (BMI) 20-35 kg/m ^2^were eligible. Subjects were excluded if physical and mental health status, including laboratory abnormalities, indicated serious or chronic illness (abnormal liver function tests (LFTs) or electrolytes, creatinine clearance < 60 mL/min, haemoglobin < 10 g/L), if they were intolerant to magnesium-containing products, taking certain medications (antacids other than proton pump inhibitors or H _2_agonists, diuretics, calcium or magnesium supplements) or planned medication changes. Subjects using hormone replacement therapy (HRT) were to have been on a stable dose for at least one month prior to screening and continue this dose through the study. Subjects on special diets (including vegan, weight loss or high protein), those with a history of frequent use of magnesium based laxatives and substance abuse (including nicotine) were excluded.

### Protocol - treatment

Of the 91 screened, 23 failed to satisfy the entry criteria and one was excluded due to poor venous access. The remaining 67 postmenopausal women were randomised in a 1:1 ratio to receive between 1500 mL and 1800 mL daily of magnesium bicarbonate supplemented spring water (650 mg/L bicarbonate, 120 mg/L magnesium, pH 8.3-8.5) (supplemented water group) or spring water without supplements (control water group) over three months (84 days). For those consuming the supplemented water, this volume resulted in a daily dose of 975 - 1170 mg bicarbonate and 180 - 216 mg of magnesium. There was no bicarbonate and negligible amounts of magnesium (< 5 mg/L) in the spring water given to the control group. Analysis of the spring water (control) was conducted by National Association of Testing Authorities (NATA) accredited (#1884) SONIC HEALTHCARE laboratory NSW and the constituents are listed in Additional file 1.

The water was supplied in 600 mL identical bottles so that subjects were blinded to the type of water they received. Subjects were at liberty to choose the time at which they consumed the water and were discouraged from drinking the study product with meals. The water could be chilled but not heated to be used in beverages such as tea or coffee, or for cooking. All study water consumed was recorded by subjects in the drink diary and bottle caps returned at each study visit to evaluate compliance, where 100% compliance was defined as daily consumption of between 1500 mL and 1800 mL of water. Subjects were requested to maintain consistency of nutritional habits and lifestyle behaviours throughout the study period.

### Measurements

Subjects were seen at Baseline and at Days 14, 42 and 84. Any changes in diet were noted. Blood and urine samples were collected at every visit to measure markers of bone turnover (PTH, 1,25-dihydroxyvitamin D and osteocalcin, urinary telopeptides, hydroxyproline) and pH. Serum fasting lipids (total cholesterol, HDL, LDL, triglycerides), serum biochemistry (albumin, alkaline phosphatase, alanine amino transferase [ALT], aspartate amino transferase [AST], bicarbonate, total bilirubin, chloride, calcium, creatine kinase, creatinine, fasting glucose, lactate dehydrogenase [LDH], magnesium, potassium, sodium, total protein, urea and uric acid) and haematology were measured at each visit. Urine was collected for 24 h for the measurement of calcium, phosphate, creatinine and free cortisol concentrations and excretion. Physical examinations were performed; including blood pressure (supine and standing); body weight recorded and adverse event reports were elicited at each visit.

All biochemical, haematology and urinalysis testing was performed by NATA accredited (#2115) Institute of Laboratory Medicine (SydPath) St Vincent's Hospital NSW.

### Statistics

The change from Baseline (Day 0) to each study visit at Days 14, 42 and 84 was compared between treatment groups for all biochemical, blood lipid, blood pressure, pH measures, haematological, urinalysis and bone turnover markers. Tables and figures present the mean and standard deviation (SD) for each parameter at baseline and at each visit. Continuous measures were compared across treatments using independent, two-sample, unpaired t-tests if the data were normally distributed, otherwise the Wilcoxon Rank Sum Test was used. Categorical measures were compared using Chi square or Fisher's Exact test. No adjustments were made for multiple testing as this was an exploratory study and it is acknowledged that there is an increased risk of making Type I errors given the number of tests performed.

Repeated measures analyses of variance were performed for all outcome variables, incorporating all visits from baseline to Day 14, Day 42 and Day 84 together with treatment group as factors in the analysis. If the p-value for the change in the variable was less than or equal to 0.10 then a generalised linear mixed model with a random intercept term (for subject) was fitted. The outcome in the model was the measure of interest at Days 0, 14, 42 and 84 and the model included the main effects of group and visit. The p-value from the test of the interaction between group and time was the result of interest and p < 0.05 was considered statistically significant.

## Results

Sixty-seven eligible subjects received at least one dose of magnesium bicarbonate supplemented spring water {supplemented water group; n = 34} or one dose of spring water without supplement {control group; n = 33}, had a valid baseline measurement and returned for at least one post-baseline visit. All analyses were carried out on this population which was identical to the safety population.

Mean (SD) age was 57 (4.4) years and 59 (5.5) years in the control and supplemented water groups, respectively. Body weight and BMI remained unchanged in both groups (Table 1) over the study period. Dietary habits in both groups were unchanged as assessed at each visit.

**Table 1:** Body weight and BMI at all visits for spring water and magnesium bicarbonate (supplemented) spring water groups

		Spring Water (n = 33)				Supplemented Spring Water (n = 34)			
	**Visit**	**Day 0**	**Day 14**	**Day 42**	**Day 84**	**Day 0**	**Day 14**	**Day 42**	**Day 84**

Weight (kg)	Mean (SD)	67.99 (11.58)	68.09 (11.76)	68.11 (12.03)	67.86 (11.54)	64.22 (8.15)	64.24 (8.32)	64.10 (8.20)	64.07 (8.61)
	
	Change from baseline (Day 0) (SD)		0.02 (1.01)	0.03 (1.40)	-0.21 (1.98)		0.02 (0.78)	-0.12 (1.36)	-0.15 (1.66)
	
	*P value					*0.126*	0.921	0.749	0.946

BMI (kg/m2)	Mean (SD)	25.05 (3.64)	25.11 (3.71)	25.11 (3.78)	25.02 (3.59)	24.16 (3.14)	24.17 (3.18)	24.12 (3.13)	24.16 (3.14)
	
	Change from baseline (Day 0) (SD)		0.010 (0.38)	0.009 (0.54)	-0.073 (0.76)		0.006 (0.29)	-0.048 (0.51)	-0.052 (0.62)
	
	*P value					*0.289*	0.991	0.681	0.930

*p-value (t-test) comparing groups at Day 0 (italics) and for change from Baseline to Day 14, Day 42 and Day 84

There were no significant group differences in all parameters measured at baseline (Day 0) (Tables 2, 3, 4, 5, Additional files 2, 3, 4, 5).

**Table 2:** Serum bone metabolic markers over the study period for spring water and magnesium bicarbonate (supplemented) spring water groups

		Spring Water (n = 33)		Supplemented Spring Water (n = 34)	
	**Visit**	**Day 0**	**Day 84**	**Day 0**	**Day 84**

Parathyroid Hormone (pmol/L)	Mean (SD)	3.85 (1.79)	4.57 (2.14)	4.24 (1.74)	4.21 (1.96)
	
	Change from baseline (Day 0) (SD)		0.72 (1.52)		-0.02 (1.65)
	
	*P value			*0.369*	**0.059**

1,25dihydroxyvitamin D (pmol/L)	Mean (SD)	120.8 (40.9)	126.8 (40.4)	118.7 (39.8)	124.3 (40.7)
	
	Change from baseline (Day 0) (SD)		7.1 (49.1)		5.6 (47.9)
	
	*P value			*0.835*	0.766

Osteocalcin	Mean (SD)	13.73 (5.44)	14.42 (5.88)	15.23 (3.89)	13.79 (3.48)
	
(μg/L)	Change from baseline (Day 0) (SD)	0.70 (4.00)	0.07 (4.00)		-1.31 (3.89)
	
	*P value			*0.207*	0.104

N-telopeptide excretion	Mean (SD)	36.7 (21.7)	40.1 (21.3)	34.1 (17.8)	35.1 (15.7)
	
nmol/mM Cr)	Change from baseline (Day 0) (SD)		3.4 (12.66)		1.0 (15.97)
	*P value			*0.588*	0.440

OH proline excretion (nmol/mM Cr)	Mean (SD)	15.5 (5.39)	17.2 (6.75)	15.5 (4.98)	15.6 (4.57)
	
	Change from baseline (Day 0) (SD)		1.6 (5.98)		0.1 (4.70)
	
	*P value			*0.971*	0.216

*p-value comparing groups at Day 0 (italics) and for change from Day 0 to Day 84

**Table 3:** Serum lipid profile over the study period for spring water and magnesium bicarbonate (supplemented) spring water groups

		Spring Water (n = 33)		Supplemented Spring Water (n = 34)	
	**Visit**	**Day 0**	**Day 84**	**Day 0**	**Day 84**

Triglyceride	Mean (SD)	1.12 (0.45)	0.95 (0.39)	0.95 (0.32)	0.92 (0.46)
	
(mmol/L)	Change from baseline (Day 0) (SD)		-0.17 (0.46)		-0.03 (0.39)
	
	*P value			*0.107*	0.528

Cholesterol	Mean (SD)	5.79 (1.6)	5.69 (1.21)	5.44 (0.78)	5.51 (0.93)
	
(mmol/L)	Change from baseline (Day 0) (SD)		-0.10 (0.63)		0.07 (0.53)
	
	*P value			*0.260*	0.484

High Density Lipoprotein	Mean (SD)	1.72 (0.50)	1.78 (0.47)	1.75 (0.32)	1.77 (0.32)
	
(mmol/L)	Change from baseline (Day 0) (SD)		0.05 (0.19)		0.02 (0.19)
	
	*P value			*0.772*	0.576

Low Density Lipoprotein	Mean (SD)	3.57 (1.38)	3.48 (1.07)	3.31 (0.90)	3.33 (0.89)
	
	Change from baseline (Day 0) (SD)		-0.08 (0.50)		0.02 (0.38)
	
	*P value			*0.364*	0.611

*p-value comparing groups at Day 0 (italics) and for change from Day 0 to Day 84

**Table 4:** Blood pressure (BP) and urinary pH over the study period for spring water and magnesium bicarbonate (supplemented) spring water groups

		Spring Water (n = 33)		Supplemented Spring Water (n = 34)	
	Visit	Day 0	Day 84	Day 0	Day 84
§SUPINE SYSTOLIC BP (mm Hg)	Mean (SD)	119.6 (13.23)	117.4 (10.73)	121.5 (13.32)	121.9 (14.08)
	
	Change from baseline (Day 0) (SD)		-2.3 (11.64)		0.4 (10.74)
	
	*P value			*0.562*	0.1606

§SUPINE DIASTOLIC BP (mm Hg)	Mean (SD)	73.1 (8.88)	72.0 (6.95)	74.1 (8.47)	73.9 (8.68)
	
	Change from baseline (Day 0) (SD)		-1.2 (7.58)		-0.2 (6.36)
	
	*P value			*0.650*	0.367

Urinary pH	Mean (SD)	6.13 (0.93)	6.17 (0.83)	6.38 (0.63)	6.66 (0.56)
	
	Change from baseline (Day 0) (SD)		0.05 (0.86)		0.28 (0.73)
	
	*P value			*0.197*	**0.018**

*p-value comparing groups at Day 0 (italics) and for change from Day 0 to Day 84

§ Standing systolic and diastolic BP showed no significant difference between two water groups (Data not shown)

**Table 5:** Serum electrolytes over the study period for spring water and magnesium bicarbonate (supplemented) spring water groups

		Spring Water (n = 33)		Supplemented Spring Water (n = 34)	
	**Visit**	**Day 0**	**Day 84**	**Day 0**	**Day 84**

Bicarbonate (mmol/L)	Mean (SD)	27.6 (1.84)	27.6 (1.60)	27.9 (1.94)	27.8 (1.93)
	
	Change from baseline (Day 0) (SD)		0.0 (2.01)		-0.1 (1.60)
	
	*P value			*0.511*	0.790

Corrected Calcium (mmol/L)	Mean (SD)	2.31(0.07)	2.29 (0.09)	2.32 (0.08)	2.31 (0.091)
	
	Change from baseline (Day 0) (SD)		-0.03 (0.07)		-0.004 (0.06)
	
	*P value			*0.778*	0.1504

Magnesium (mmol/L)	Mean (SD)	0.86 (0.06)	0.86 (0.06)	0.87 (0.05)	0.90 (0.052)
	
	Change from baseline (Day 0) (SD)		0.006 (0.04)		0.03 (0.03)
	
	*P value			*0.320*	**0.006**

*p-value comparing groups at Day 0 (italics) and for change from Day 0 to Day 84

The most common pre-existing conditions in both groups were self-reported drug hypersensitivity, osteoarthritis and lipid disorders for which subjects used hypolipidaemics, anti-inflammatory and antirheumatic products and analgesics. The compliance rate was high in both groups {28 (84.8%) in the control and 29 (85.3%) and supplemented water groups} according to daily diary records. The 1500 mL -1800 mL of water volume was well tolerated by all subjects.

### Outcome Analyses

There were no statistically significant differences in change between the supplemented water group and the water control group from baseline to Day 14, 42 and 84 in any of the measures of bone mineral turnover (n-telopeptides, hydroxyproline, osteocalcin and 1,25-dihydroxyvitamin D) (Table 2, Additional file 2) or in cardiovascular parameters of serum lipid profile measures (triglycerides, LDL HDL and total cholesterol) (Table 3, Additional file 3) and blood pressure (Table 4, Additional file 4). Measurements of bicarbonate, calcium, creatinine, sodium (Table 5, Additional file 5) and most serum biochemistry also did not show significant differences between the two groups remaining within normal ranges (data not shown). Haematology parameters remained unaltered through study period for both water groups (data not shown).

**Figure 1:**
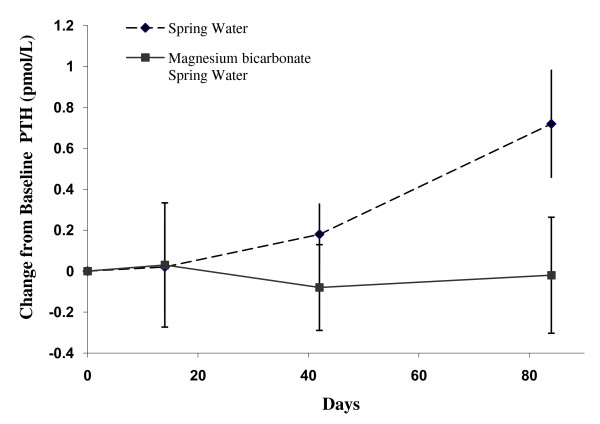
**Change in serum Parathyroid Hormone (PTH) levels in the Magnesium supplemented Bicarbonate spring water and spring water (control) groups at Day 14, 42 and 84**. Values are mean ± standard error by treatment group and study day

The concentration of serum PTH increased from Day 0 (3.85 pmol/L) to Day 84 (4.57 pmol/L) with control water consumption compared to supplemented water for which the concentration remained relatively constant (4.24 and 4.21 pmol/L, respectively). There was a trend towards a difference in the change in PTH concentration between the two groups from Day 0 to Day 84 (0.74 pmol/L; 95% Confidence Interval (CI): -0.03 to 1.52 pmol; p = 0.059) (Figure 1, Table 2). There was no significant difference in the change in PTH concentration between the two groups at Day 0 to Day 14 or Day 42 (see Additional file 2).

**Figure 2:**
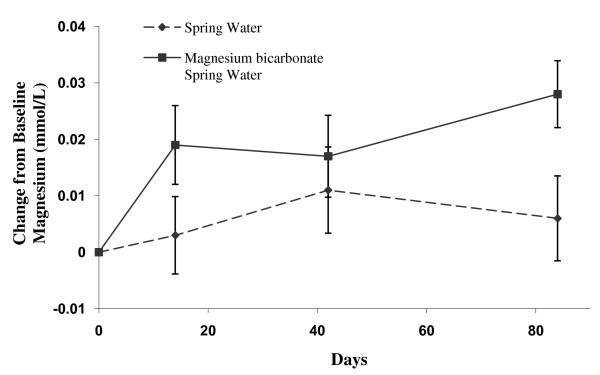
**Change in serum Magnesium in the Magnesium Bicarbonate supplemented spring water and spring water (control) groups at Day 14, 42 and 84**. Values are mean ± standard error by treatment group and study day

The serum concentration of magnesium increased with the intake of the supplemented water compared to that of control water intake {0.022 mmol/L (95% CI: 0.003 to 0.041 mmol/L)} (Table 5, Additional file 5). The difference between the two groups in the change from Day 0 to Day 84 in the serum concentrations of magnesium was statistically significant (p = 0.027) (Figure 2, Table 5). When data from all time points were analysed using a repeated measures analysis of variance the difference between groups in the serum concentrations of magnesium remained statistically significant (p = 0.015). Serum potassium was also increased by Day 84 with the intake of supplemented water (p = 0.054) (Additional File 5).

**Figure 3:**
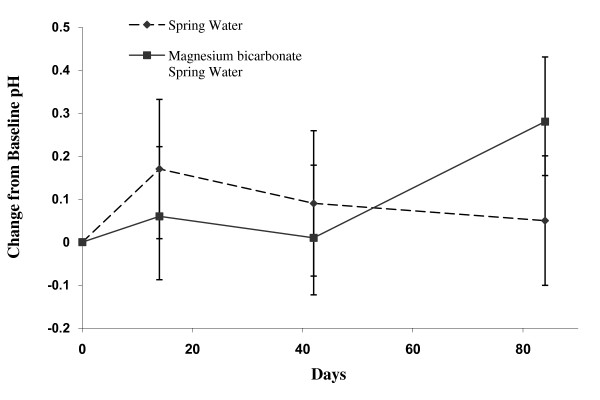
**Change in Urinary pH in Magnesium Bicarbonate supplemented water and spring water (control) groups at Day 14, 42 and 84**. Values are mean ± standard error by treatment group and study day

Consumption of the supplemented water also significantly increased the urinary pH at 84 days (p = 0.0182) (Figure 3, Table 4). The venous pH remained unaltered (Additional file 4) over the study period. There were no other statistically significant differences in change from Day 0 to Day 84 comparing control and supplemented water groups in any of the other 24-hour urinary analytes (data not shown).

### Safety Evaluation

The most common adverse event considered to be related to the water consumption was diarrhoea in five (15.2%) and eight (23.5%) subjects in the control and supplemented water groups, respectively. No subject suffered a serious adverse event during this study.

## Discussion

The present study did not show any effect of the consumption of spring water supplemented with magnesium and bicarbonate, as part of a daily routine including usual dietary habits, on clinically relevant bone parameters or cardiovascular indices over a period of 12 weeks.

The bicarbonate component was sufficient to change urinary pH values, statistically significantly by Day 84, as an indicator of acid base balance [21]. The blood bicarbonate as an indirect parameter was not different between the groups. Other studies of mineral waters containing bicarbonate at greater concentrations (approximately 1 - 2 g/L) than employed here (650 mg/L) have shown a decrease in bone resorption markers in young women [22,23] and a lowering of markers of atherosclerosis in postmenopausal women [24,25]. It is likely that the level of 650 mg/L bicarbonate in the present study was too low to elicit an effect on bone metabolic and cardiovascular parameters over a short period. In addition, the consumption of a high calcium mineral water has shown that bone modelling parameters were only affected when the study population had low dietary mineral intakes [26].

The present study showed that consumption of magnesium in alkaline aqueous formulation (magnesium bicarbonate supplemented spring water) provided a source of orally available magnesium as evidenced by the increase in serum magnesium concentrations over the 12 weeks of the study period. Other metabolic studies have shown that magnesium mineral waters with magnesium content (110 mg/L and 282 mg/L) similar to this study water of 120 mg/L could be easily absorbed and retained in healthy men and women [27,28]. It has been suggested that absorption becomes impaired with increasing age [29], thus the magnesium bicarbonate supplemented spring water could provide a source of magnesium particularly in older women. However, since this study did not measure dietary magnesium intake or excreted magnesium, the absolute contribution of the magnesium from the supplemented spring water remains unknown.

It is acknowledged that other parameters measured in this study will be influenced by the composition of diet and energy loads. Accordingly the rise in serum potassium by Day 84 with the consumption of the supplemented water remains observational. Thus, despite the requested maintenance of usual dietary habits by all subjects in the study, the lack of dietary records in the study population is a limitation of this exploratory investigation.

In this present study, PTH concentrations remained stable over 12 weeks in the magnesium bicarbonate water group. This finding is consistent with other studies in normal subjects where intake of oral alkaline [28] and magnesium [30] loads do not alter PTH levels in the short term. It remains to be determined, if the trend to increase in PTH with the consumption of spring water alone becomes sufficient to potentiate the action of PTH on bone metabolism in a clinically meaningful way and if magnesium bicarbonate supplemented spring water has favourable effects on PTH concentrations and bone metabolism with longer-term consumption.

In summary, these results suggest that the consumption of magnesium rich bicarbonate spring water may provide a source of magnesium to contribute to total magnesium intake. Changes in urinary pH may, in the longer term, lead to improved acid base balance. Longer term studies, that include assessment of diet, urinary volume and mineral excretion, are required to determine the clinical relevance of these findings.

## Conclusions

Short term consumption of magnesium bicarbonate supplemented spring water increased serum magnesium and urinary pH in postmenopausal women. There was no effect on bone metabolism or cardiovascular risk factors. Whether the former changes are of clinical relevance with respect to various processes, such as those associated with aging which have been linked to magnesium insufficiency and/or acidosis, remains to be determined by further research.

## Competing interests

Dr Beckett is a Director of Unique Global Possibilities Medical Pty Ltd which sponsored the trial. All other authors declare no competing interests.

## Authors' contributions

ROD developed the study design and plan as well as oversaw the drafting of and was a major contributor to the manuscript. WL participated in the study design, provided input in the clinical study protocol and contributed to the writing of the manuscript. LMRT provided input in the clinical study protocol, provided input into and oversight of the Clinical Study Report, drafted, coordinated and finalised the writing of the manuscript. PM performed the statistical data analysis and provided input into the Clinical Study Report. RJB conceived of the study and contributed to the writing of the manuscript. KMW participated in the study design and contributed to the writing of the manuscript. All authors read and approved the manuscript.

## Supplementary Material

Additional file 1 Analysis of *Spring Water (control). *spring (Peats Ridge) water is commercially registered by the NSW government. Click here for file

Additional file 2 **Serum bone metabolic markers at all visits for two water treatment groups**. Click here for file

Additional file 3 **Serum lipid profile at all visits for two water treatment groups**. Click here for file

Additional file 4 **Blood pressure (BP) and pH at all visits for two water treatment groups**. Click here for file

Additional file 5 **Serum biochemistry at all visits for two water treatment groups**. Click here for file
