# Supplementary material for: A double-blind, placebo-controlled study of the short term effects of a spring water supplemented with magnesium bicarbonate on acid/base balance, bone metabolism and cardiovascular risk factors in postmenopausal women
Source: BMC Res Notes. 2010 Jun 28;3:180. doi: 10.1186/1756-0500-3-180 (PMC2908636; doi:10.1186/1756-0500-3-180)
Supplement: Additional file 1 — Analysis of *Spring Water (control). *spring (Peats Ridge) water is commercially registered by the NSW government. [file 1756-0500-3-180-S1.PDF]

Additional file 1. Analysis of *Spring Water (control)

Chemical	mg/Litre	Detection
**Aluminium**	0.5	
**Arsenic**	<0.01	No
**Barium**	<0.1	No
**Borate**	<0.1	No
**Bicarbonate**	<1	No
**Calcium**	0.4	
**Cadmium**	<0.001	No
**Chemical oxygen demand**	<2	No
**Chlorine (free)**	<0.01	No
**Chromium**	<0.01	No
**Copper**	<0.01	No
**Cyanide**	<0.01	No
**Fluoride**	<0.01	No
**Iron (total)**	0.04	
**Lead**	<0.01	No
**Manganese**	<0.01	No
**Magnesium**	3.3	
**Mercury**	<0.001	No
**Nitrate**	12	
**Nitrite**	<0.1	No
**Potassium**	0.6	
**Selenium**	<0.05	No
**Sodium**	13	
**Sulphate**	9	
**Sulphide**	<0.01	No
**Zinc**	<0.01	No
**Conductivity**	95 us/cm	
**PH**	4.2	

*spring (Peats Ridge) is commercially registered by the NSW government
