# Supplementary material for: A double-blind, placebo-controlled study of the short term effects of a spring water supplemented with magnesium bicarbonate on acid/base balance, bone metabolism and cardiovascular risk factors in postmenopausal women
Source: BMC Res Notes. 2010 Jun 28;3:180. doi: 10.1186/1756-0500-3-180 (PMC2908636; doi:10.1186/1756-0500-3-180)
Supplement: Additional file 2 — Serum bone metabolic markers at all visits for two water treatment groups. [file 1756-0500-3-180-S2.PDF]

Additional file 2. Serum bone metabolic markers at all visits for two treatment group

		Spring Water (n = 33)				Supplemented Spring Water (n= 34)			
	**Visit**	**Day 0**	**Day 14**	**Day 42**	**Day 84**	**Day 0**	**Day 14**	**Day 42**	**Day 84**
Parathyroid Hormone (pmol/L)	Mean (SD)	3.85 (1.79)	3.87 (1.75)	4.03 (1.73)	4.57 (2.14)	4.24 (1.74)	4.26 (1.73)	4.16 (1.59)	4.21 (1.96)
	Change from baseline (Day 0) (SD)		0.02 (1.28)	0.18 (0.87)	0.72 (1.52)		0.03 (1.77)	-0.08 (1.22)	-0.02 (1.65)
	*P value					*0.369*	0.645	0.488	**0.059**
1 25 Dihydroxyvitamin D (pmol/L)	Mean (SD)	120.8 (40.9)	112.8 (41.9)	113.5 (43.5)	126.8 (40.4)	118.7 (39.8)	116.0 (32.7)	109.9 (34.4)	124.3 (40.7)
	Change from baseline (Day 0) (SD)		-7.3 (47.3)	-6.8 (46.6)	7.1 (49.1)		-2.7 (42.5)	-8.7 (44.4)	5.6 (47.9)
	*P value					*0.835*	0.716	0.716	0.766
Osteocalcin (µg/L)	Mean (SD)	13.73 (5.44)	14.53 (6.09)	15.60 (6.64)	14.42 (5.88)	15.23 (3.89)	14.80 (3.45)	15.66 (4.23)	13.79 (3.48)
	Change from baseline (Day 0) (SD)		0.80 (4.10)	1.87 (4.51)	0.70 (4.00)		-0.15 (3.07)	0.79 (3.18)	-1.31 (3.89)
	*P value					*0.207*	0.511	0.393	0.104
N-telopeptide excretion (nmol/mM Cr)	Mean (SD)	36.7 (21.7)	38.8 (26.8)	32.1 (18.9)	40.1 (21.3)	34.1 (17.8)	32.3 (12.1)	31.9 (13.6)	35.1 (15.7)
	Change from baseline (Day 0) (SD)		2.6 (13.25)	-4.6 (14.52)	3.4 (12.66)		-1.8 (14.52)	-2.1 (13.52)	1.0 (15.97)
	*P value					*0.588*	0.153	0.714	0.440
OH proline excretion (nmol/mM Cr)	Mean (SD)	15.5 (5.39)	16.3 (6.00)	15.5 (7.05)	17.2 (6.75)	15.5 (4.98)	15.1 (4.09)	14.6 (4.01)	15.6 (4.57)
	Change from baseline (Day 0) (SD)		0.8 (2.99)	-0.1 (6.00)	1.6 (5.98)		-0.4 (4.36)	-0.9 (5.02)	0.1 (4.70)
	*P value					*0.971*	0.171	0.510	0.216

* p-value comparing groups at Day 0 (italics) and for change from Day 0 to Day14, Day 42 and Day 84
