# Supplementary material for: A double-blind, placebo-controlled study of the short term effects of a spring water supplemented with magnesium bicarbonate on acid/base balance, bone metabolism and cardiovascular risk factors in postmenopausal women
Source: BMC Res Notes. 2010 Jun 28;3:180. doi: 10.1186/1756-0500-3-180 (PMC2908636; doi:10.1186/1756-0500-3-180)
Supplement: Additional file 3 — Serum lipid profile at all visits for two water treatment groups. [file 1756-0500-3-180-S3.PDF]

Additional file 3. Serum lipid profile at all visits for two treatment groups

		Spring Water (n = 33)				Supplemented Spring Water (n= 34)			
	**Visit**	**Day 0**	**Day 14**	**Day 42**	**Day 84**	**Day 0**	**Day 14**	**Day 42**	**Day 84**
Triglyceride (mmol/L)	Mean (SD)	1.12 (0.45)	1.12 (0.55)	1.07 (0.47)	0.95 (0.39)	0.95 (0.32)	0.89 (0.34)	0.85 (0.48)	0.92 (0.46)
	Change from baseline (Day 0) (SD)		0.00 (0.33)	-0.05 (0.38)	-0.17 (0.46)		-0.05 (0.25)	-0.10 (0.35)	-0.03 (0.39)
	*P value					*0.107*	0.28	0.33	0.528
Cholesterol (mmol/L)	Mean (SD)	5.79 (1.6)	5.85 (1.45)	5.83 (1.5)	5.69 (1.21)	5.44 (0.78)	5.42 (0.83)	5.51 (0.90)	5.51 (0.93)
	Change from baseline (Day 0) (SD)		0.06 (0.42)	0.04 (0.68)	-0.10 (0.63)		-0.01 (0.46)	0.08 (0.51)	0.07 (0.53)
	*P value					*0.260*	0.258	0.967	0.484
High Density Lipoprotein (mmol/L)	Mean (SD)	1.72 (0.50)	1.76 (0.49)	1.75 (0.51)	1.78 (0.47)	1.75 (0.32)	1.79 (0.36)	1.76 (0.29)	1.77 (0.32)
	Change from baseline (Day 0) (SD)		0.04 (0.15)	0.03 (0.18)	0.05 (0.19)		0.06 (0.14)	0.02 (0.15)	0.02 (0.19)
	*P value					*0.772*	0.530	0.875	0.576
Low Density Lipoprotein (mmol/L)	Mean (SD)	3.57 (1.38)	3.58 (1.29)	3.60 (1.29)	3.48 (1.07)	3.31 (0.90)	3.22 (0.85)	3.37 (0.87)	3.33 (0.89)
	Change from baseline (Day 0) (SD)		0.02 (0.36)	0.03 (0.61)	-0.08 (0.50)		-0.08 (0.42)	0.06 (0.29)	0.02 (0.38)
	*P value					*0.364*	0.156	0.975	0.611

* p-value comparing groups at Day 0 (italics) and for change from Day 0 to Day14, Day 42 and Day 84
