# Supplementary material for: A double-blind, placebo-controlled study of the short term effects of a spring water supplemented with magnesium bicarbonate on acid/base balance, bone metabolism and cardiovascular risk factors in postmenopausal women
Source: BMC Res Notes. 2010 Jun 28;3:180. doi: 10.1186/1756-0500-3-180 (PMC2908636; doi:10.1186/1756-0500-3-180)
Supplement: Additional file 4 — Blood pressure (BP) and pH at all visits for two water treatment groups. [file 1756-0500-3-180-S4.PDF]

Additional file 4. Blood pressure (BP) and pH at all visits for two treatment groups

		Spring Water (n = 33)				Supplemented Spring Water (n= 34)			
	**Visit**	**Day 0**	**Day 14**	**Day 42**	**Day 84**	**Day 0**	**Day 14**	**Day 42**	**Day 84**
SUPINE § SYSTOLIC BP	Mean (SD)	119.6 (13.23)	116.0 (13.50)	118.7 (11.32)	117.4 (10.73)	121.5 (13.32)	119.4 (12.27)	120.6 (13.92)	121.9 (14.08)
(mm Hg)	Change from baseline (Day 0) (SD)		-3.6 (12.43)	-1.0 (12.76)	-2.3 (11.64)		-2.1 (10.92)	-0.9 (11.24)	0.4 (10.74)
	*P value					*0.562*	0.368	0.724	0.161
SUPINE § DIASTOLIC BP	Mean (SD)	73.1 (8.88)	71.4 (9.71)	71.3 (8.57)	72.0 (6.95)	74.1 (8.47)	72.9 (8.02)	73.3 (8.27)	73.9 (8.68)
(mm Hg)	Change from baseline (Day 0) (SD)		-1.8 (6.91)	-1.8 (6.58)	-1.2 (7.58)		-1.1 (7.11)	-0.8 (6.41)	-0.2 (6.36)
	*P value					*0.650*	0.576	0.347	0.367
Venous Blood pH	Mean (SD)	7.37 (0.024)	7.37 (0.03)	7.37 (0.03)	7.37 (0.02)	7.37 (0.034)	7.37 (0.032)	7.37 (0.028)	7.38 (0.043)
	Change from baseline (Day 0) (SD)		0.002	0.000	0.000		0.003	0.001	0.007
	*P value					*0.839*	0.771	0.875	0.264
Urinary pH	Mean (SD)	6.13 (0.93)	6.29 (0.80)	6.24 (0.88)	6.17 (0.83)	6.38 (0.63)	6.44 (0.71)	6.40 (0.80)	6.66 (0.56)
	Change from baseline (Day 0) (SD)		0.17 (0.93)	0.09 (0.97)	0.05 (0.86)		0.06 (0.86)	0.01 (0.77)	0.28 (0.73)
	*P value					*0.197*	0.740	0.757	0.018

* p-value comparing groups at Day 0 (italics) and for change from Day 0 to Day14, Day 42 and Day 84

§ Standing systolic and diastolic BP showed no significant difference between two water groups (Data not shown)
